# *“We assist the health system doing the work that should be done by others” –* a qualitative study on experiences of grassroots level organizations providing refugee health care during the 2015 migration event in Germany

**DOI:** 10.1186/s12913-022-07683-2

**Published:** 2022-03-07

**Authors:** Stephan Brenner, Vincent Lok

**Affiliations:** Heidelberg Institute of Global Health, Ruprecht-Karls Universität Heidelberg, Im Neuenheimer Feld 130.3, 69120 Heidelberg, Germany

**Keywords:** Refugee health care, Germany, Non-governmental actors, Qualitative research, Migrant crisis

## Abstract

**Background:**

In Germany, the 2015 mass displacement and resulting population migration exposed regulatory and structural shortcomings with respect to refugee healthcare provision. Existing research on Germany’s crisis response has largely focused on the roles played by public and health system actors. The roles and contributions of non-governmental actors operating at the grassroots level have so far been given little attention. The purpose of this qualitative study was to explore the involvement of grassroots level actors with refugee healthcare provision in Germany.

**Methods:**

In 2017, we conducted in-depth interviews with 13 representatives of different non-governmental organizations providing refugee healthcare provision in Germany. This included humanitarian relief organizations operating at the grassroots level that offer various forms of medical and psychological care. Transcribed interview content was analyzed using both deductive and inductive coding approaches.

**Results:**

Grassroots level involvement changed over the course of the reporting period. During the initial emergency response, locally organized groups supported federal states and municipalities to guarantee the provision of legally defined refugee healthcare. During the following less acute phase, grassroots organizations attended to health needs of refugees the public health system was unable to address due to legal or structural limitations. In the subsequent integration phase, grassroots organizations shifted their relief focus towards care for the most vulnerable among refugees, including rejected asylum seekers and undocumented migrants with no or limited health coverage, as well as for those suffering from mental health problems.

**Conclusion:**

Grassroots actors perceived their contributions largely as addressing those bottlenecks that resulted from healthcare restrictions imposed by German refugee legislation. Such bottlenecks could be addressed by offering those medical services for free that otherwise were not covered by law. Further, volunteers contributed to closing existing information and communication gaps between public actors, serving as intermediaries between public officials, healthcare providers, and refugee patients. To increase Germany’s efficiency and preparedness with respect to refugee healthcare, more integrated approaches at the local level, patient-centered interpretation and implementation of refugee law, and a stronger focus on post-traumatic mental health disorders should be considered.

**Supplementary Information:**

The online version contains supplementary material available at 10.1186/s12913-022-07683-2.

## Background

### The 2015 refugee situation and humanitarian relief in Europe

In 2015, Europe experienced one of the biggest migration events in recent decades. The vast movements of displaced people and refugees – a majority fleeing the military conflict in Syria – produced a state of humanitarian emergency in many European countries. Both national and international humanitarian relief organizations partnered with governments, donors, private sector actors, and other stakeholders to support countries in their domestic response efforts [1]. Within the wider European context, international humanitarian support focused largely on transit countries along the Balkan route crossing Southeast Europe [2]. The destination and host countries across Central Europe received far less international relief support, resulting in emergency response strategies that were far less formal and largely driven by local public and non-governmental actors at the grass-roots level [2].

In any humanitarian crisis, the provision of healthcare to crisis-affected population groups is of central priority but also a major challenge. Since long, and especially in the context of mass displacements, humanitarian relief organizations have been key in guaranteeing refugees access to basic needs, including healthcare [3]. In contrast, many Central European host countries, including Germany, have restrictions on the access to publicly funded healthcare for newly arriving refugees [4]. In 2015 Germany, civil society, non-governmental organizations, and other non-state actors had to step in to ensure refugees’ access to essential healthcare where this was limited or denied by health system regulations [2].

### Background on refugee healthcare in Germany

Although Germany’s health system has achieved universal health coverage, certain vulnerable groups, such as impoverished or homeless citizens and undocumented or illegal migrants, still lack access to essential healthcare [5, 6]. Traditionally, non-governmental actors at the grassroots, such as faith-based charities or locally organized associations, have offered health-related services within or outside the formal health system to individuals and families who otherwise cannot access care or might face legal repercussions doing so within the public system [7]. Locally, such services are commonly provided for free in health clinics staffed by both medical and non-medical volunteers.

German immigration law requires that state and local governments temporarily (i.e., for a period of 15 months) limit refugees’ access to publicly financed healthcare. For newly arriving refugees, only basic services, such as emergency care, pregnancy-related care, and care related to infection prevention measures (i.e. vaccinations, screening and treatment of public health relevant communicable diseases) are publicly covered [8]. However, in 2015, many refugees fleeing conflict and war zones required better tailored forms of medical and psychological care not considered essential by law [9].

With the scientific literature on refugee healthcare steadily expanding, the various shortcomings in Germany’s refugee policies during the 2015 emergency response have become more visible. For instance, many municipalities faced logistical and administrative barriers to even offering critical health services already covered under immigration law [10, 11]. Further, non-covered health services commonly needed by newly arriving migrants included basic dental, chronic, and mental care [12–14]. In facing access restrictions to essential care services, many refugees instead had to seek care directly from hospital-based emergency departments outside the primary care sector [15–17].

### Objective of this study

The existence of bottlenecks in the operationalization of refugee healthcare provision under Germany’s publicly funded health system in 2015 has been pointed out and widely examined in the public health literature. The roles of non-governmental actors and their contributions to Germany’s 2015 emergency response have so far been overlooked. However, a closer look at these grassroots activists offers a so far missing perspective into the accomplishments and challenges experienced by these actors during the 2015 crisis response. This focus is of interest, since international relief organizations did not play a coordinating or otherwise dominant role in coordinating or executing the response strategies developed in the context of economically more stable countries in Central Europe [18].

This study therefore explores the involvement of grass-roots level relief organizations in providing refugee healthcare during the 2015 migrant crisis in Germany. In conducting a series of qualitative interviews with non-governmental actors, our main objective was to understand the contributions of grassroots level activists to Germany’s overall emergency response, to expand the current evidence base with respect to the organization and provision of refugee healthcare, and to identify additional bottlenecks overlooked by current research.

## Methods

### Design & Sampling

This cross-sectional qualitative study uses in-depth interviews conducted with representatives of various German non-governmental actors active in the organization and/or provision of refugee healthcare at the grassroots level in 2015. We purposefully included participants meeting the following characteristics: representatives of non-governmental organizations (defined as one of the following: associations, charities, humanitarian or relief agencies – both denominational or non-denominational – with representation at the grassroots level), operational in Germany (i.e. any of the 16 federal states), active since at least 2015 (i.e. start of refugee situation in Germany), directly involved in the organization or provision of any form of refugee healthcare (e.g. facilitation of free care, health counselling, medical or psychological care provision). We explicitly excluded any governmental agencies or their representatives, as well as volunteer-based projects or civil-society initiatives without a clear institutional or organizational structure (i.e., not listed as an official formation).

In identifying and recruiting participants, we first prepared a list of potential actors based on an extensive internet search using our inclusion criteria. This list was further expanded after consulting individuals actively involved with refugee healthcare in our region (i.e., Southwest Germany) and by each interviewed participant. The final list included a total of 77 identified associations, groups, or individuals meeting our inclusion criteria. We were able to contact 70 enlisted grassroots level activist groups or individuals by email (for seven no contact information could be identified) and provided them with background on the study objective, interview process, and research team. Of these, 36 (51.4%) responded to our repeated contact attempts, while only 13 (17.1%) decided to participate in our interviews. Reasons for non-participation included: decline to participate in an interview in 19 instances, inability to be interviewed in English in two instances, and inability to identify a suitable interview date and time in two instances.

### Data collection

Interviews were conducted between June and November 2017 (i.e., about two years after the initial emergency response). Each organization or group was asked to identify a representative most knowledgeable of the respective organization’s or group’s operational involvement with refugee healthcare. These representatives were then directly invited to participate in an interview. Informed written consent was obtained from each representative prior to interview start. VL conducted all interviews using an interview guide including potential prompts developed for this study by both authors (see Additional File 1 for more details). Interviews were conducted in English either by phone or video call with each individual respondent. All interviews were audio recorded and lasted between 45 to 90 minutes. Each interview explored four main topics: general background (organizational type, operational framework, size, roles, and responsibilities prior to 2015); roles and contributions with respect to refugee health care during and following the 2015 events; bottlenecks in publicly provided refugee healthcare and to what extent these were addressed; and changes in roles and contributions with respect to observed political or societal adjustments over time.

### Data analysis

All interviews were transcribed verbatim. Content analysis using NVivo 11 occurred in two steps: an initial deductive approach using pre-determined codes along the topics outlined in the interview guide (provided as Additional File 1); this was then followed by a series of inductive coding rounds to identify additional themes. This information was further supplemented by the interviewer notes prepared by VL during each interview. Both authors conducted their analyses separately. Emerging codes and themes were compared, discussed, and later combined during an iterative review process. Both authors identify as males and worked as researchers at the Heidelberg Institute of Global Health (HIGH) at the time this study was conducted. SB holds an MD and an MPH degree and is a senior researcher with 10+ years of experience in both quantitative and qualitative methodologies. His research focuses on universal health coverage and the evaluation of health systems mechanisms countries apply to address existing coverage gaps. VL holds a MSc degree and worked as a junior researcher on this study. He has previously worked for several years with different humanitarian non-governmental organizations in various international refugee settings. In analyzing the data, both researchers attempted to reflect the perspective of non-governmental actors and their interactions with both their client population as well as other actors within the health system. The authors further attempted to capture and present changes in political and regulatory environments related to the migration wave from the perspective of these actors.

## Results

We structured the results section into three parts. First, we present the background characteristics of respondents and their respective organizations. Next, we present findings on respondents’ contributions to refugee healthcare throughout the course of the emergency response. Lastly, we present key lessons and suggestions shared by respondents to inform future improvements in organizing refugee healthcare in Germany.

Some of the content presented in the following text and supporting quotes refers to specific terminologies describing legal aspects of the asylum application process, as these pertain to refugee healthcare entitlements. Positioning our findings within this more technical context, we ensured to explain all relevant peculiarities in sufficient detail. However, if the reader should require additional background information, we kindly refer to Table 1 which provides some more detail together with key references.

**Table 1:** Summary of specific definitions and terminology related to refugee healthcare in Germany

1.1 **Legal definitions of migrants and refugees in Germany** Recognizing international law, Germany defines **“migrants”** as persons leaving their homeland on their own accord in search of better prospects in life; **“refugees”** are defined as persons forced to flee their homeland as a result of external influences (e.g., persecution, war, conflict, or other crimes against humanity) [19]. The ***German Residence Act*** (Aufenthaltsgesetz) requires migrants to obtain a residence title (e.g., residence visa, work permit, permanent residence permit) [20]. So-called **“illegal or un-documented migrants"** refer to persons residing in Germany without or beyond the duration of their residence title, or those whose asylum application was denied. Migrants do not meet criteria for asylum (but specific arrangements exist for migrants from other EU countries).	
1.2 **Asylum application in Germany** The ***German Asylum Act*** (Asylgesetz) grants protection in form of asylum to refugees [21]. Refugees can register as “***asylum seekers*** *”* and apply for asylum through various official institutions in Germany or at its borders. Eligibility for asylum is decided by the ***Federal Office for Migration and Refugees*** (Bundesamt für Migration und Flüchtlinge, or BAMF). Positive asylum decisions fall into one of four protection categories: entitlement to asylum (i.e., persons persecuted on political grounds), refugee protection (i.e., persons persecuted by non-state players), subsidiary protection (i.e., persons with a presumed risk of serious harm in their country of origin), or national ban on deportation (i.e., person that cannot safely return to the country-of-origin due to risks of life or health). A negative decision (rejection) requires a refugee to leave Germany, which can be temporarily suspended if obstacles to deportation exist.	
1.3 **Healthcare coverage of asylum seekers and applicants in Germany** Healthcare entitlements of asylum seekers are defined in the ***Asylum Seekers Benefits Act*** (Asylbewerberleistungsgesetz), which limits publicly funded health coverage to only loosely defined health problems for a period of 15 months after arriving to Germany, and include: medical treatments of acute pain, medical care necessary to recover from or to improve or alleviate ill health, as well as pregnancy-related care and vaccinations [8]. After 15 months – or once asylum applicants receive a positive decision on their protection status – healthcare coverage includes the same entitlements as for German citizens enrolled under Social Health Insurance (SHI). During these 15 months, healthcare-related costs of asylum seekers incurred for entitled services are covered through public welfare funds administered by the local **social welfare offices**. Initially, asylum-seekers are assigned to reception centers organized by federal states and municipalities responsible for providing food, board, and other health and social benefits. Primary medical care in reception centers is provided by clinics located in these centers. Outside reception camps, healthcare is to be provided by health professionals working within the health system (i.e., general practitioners, specialists, hospitals, pharmacies, etc.) and accordingly reimbursed by the state or municipality under which an asylum seeker or applicant is registered. Prior to 2015 and in order to reimburse healthcare costs, most federal states required asylum applicants within the initial 15-month period to first contact their local social welfare office to obtain a **treatment voucher** (Behandlungsschein) prior to be treated by a medical provider [22]. From 2015 onwards, increasingly more federal states opted to introduce an **electronic health card** system to replace treatment vouchers. This system is closely aligned to the access and reimbursement processes established within the SHI system.	
1.4 **Additional legal regulations related to the asylum-seeking process after 2015** Political responses to the 2015 migration wave resulted in the passing of new or amendments to existing immigration law. In October 2015, the ***Accelerated Asylum Procedures Act*** (Asylverfahrensbeschleunigungsgesetz) affirmed the obligation of the German SHI to cover healthcare costs of entitled asylum applicants and allowed federal states to adopt the electronic health card system to replace the voucher system [23]. Further, this Act recategorized a number of countries previously considered unsafe (e.g., countries along the Balkan Route) as “safe states”, thus limiting the legal right to asylum in Germany for migrants of or passing through these countries. In March 2016, the ***Act for the Introduction of Expedited Asylum Procedures*** (Gesetz zur Einführung beschleunigter Asylverfahren) or “Asylpaket 2” limited health-related reason to include life-threatening conditions only in order to justify the halt of deportations of rejected asylum applicants [24]. As a result, diseases such as post-traumatic stress disorders (PTSD) are now no longer considered a legal obstacle to a pending deportation. Further, individuals with pending deportations are now presumed to be of adequate health unless they can provide a medical certificate prepared by a qualified physician addressing specific criteria (i.e., actual circumstances that led to such medical assessment, diagnostic methods used, the severity of the disease, and potential negative health effects in case of deportation).	

### Characteristics and operational backgrounds of study participants

#### Key characteristics of interview sample

Tables 2 and 3 outline key characteristics of respondents and their respective organizations. Overall, respondent backgrounds were rather diverse with respect to their roles and responsibilities. Three represented local associations, which were founded, existed, and operated only within their local municipal or regional context. All others represented local chapters of larger, often nationwide, networks offering various refugee health activities. Local chapters operated largely independently from their respective headquarters, each usually developing their individual grassroots approach in response to their specific local context. Local associations and local chapters of larger network organizations did not exhibit substantial differences in their operational or ideological arrangements. The one noticeable difference we observed was that local chapter representatives had often broader knowledge or awareness of differences in refugee health approaches across jurisdictions or states, as their larger networks allowed a more instant exchange of information and ideas. One feature fully underrepresented in our sample are grassroots organizations operating in the new German states. Among professions reported by respondents, medical backgrounds were most common as many of the local chapters were organized or steered by medical students.

**Table 2:** Overview key characteristics of interview respondents

Characteristics interview respondents	Distribution (Total ***N*** = 13)	
Role or responsibilities within organization	Chairperson, director	2
	Founder, co-founder	3
	Active member	5
	Project coordinator, project manager	3
Professional background	Medical (physician, student)	8
	Psycho-social (psychologist, social worker, educator)	2
	Other	3
Gender	Female	7
	Male	6

**Table 3:** Overview key characteristics of organizations represented by interview respondents

Organizational characteristics	Distribution (Total ***N =*** 13)	
Organizational form	Local chapters of national or international networks of non-governmental organizations.	10
	Locally established associations.	3
Organizational experience	Operational prior to 2015.	9
	Operational since 2015 or shortly after.	4
Organizational mission	Support access to healthcare within existing health system (including referral within local provider network, translator support, financial coverage on case-by-case basis).	7
	Provision of different types of medical care to underserved or uninsured populations.	4
	Provision of psychosocial support, counselling, social integration.	2
Staffing model	Volunteers (professionals, students, non-professionals).	8
	Professionals reimbursed for time worked (e.g., consultants, physicians, counselors).	2
	Employed staff.	1
	Mix of employed and volunteer staff.	2
Size of active staff at local level	10 or less	1
	11–20	4
	21–30	3
	31–40	2
	100 or more	3
Main funding source	Partially of fully publicly funded.	4
	Fully privately funded (donations, charitable contributions, or similar).	9
Federal state (Bundesland) of operation and/or headquarter location	Baden-Württemberg	3
	Bavaria	3
	Berlin	1
	Lower Saxony	1
	North Rhine-Westphalia	2
	Rhineland-Palatinate	3

#### Main role of non-governmental organizations before the refugee situation

Prior to 2015, grassroots activists mainly offered local support to those population groups with limited or no insurance coverage (i.e., uninsured German or European Union citizens, the homeless, undocumented migrants, but also asylum seekers). Prior to 2015, the proportion of asylum seekers among their clientele used to be quite small. Besides the organization and facilitation of access to free healthcare, additional activities also included advocating for their clients’ right to receive their entitled health and non-health benefits in cases where this was delayed or denied.

*“We try to provide access to the same sort of healthcare everyone should have. We don’t treat our clients and we don’t give them any medical advice. But we try to accompany them to professional healthcare providers, decide what kind of doctor a patient should see, at what time, and how to best provide access.”* (local chapter of national network, active since 2015).

Most grassroots level activists organized access to free care through local networks of volunteering healthcare providers offering to treat non-covered patients for free. These volunteer networks consisted mainly of general practitioners, outpatient clinics, or pharmacies. Clinical services commonly offered included family and pediatric care, in some instances also gynecological and midwifery services, or dental care. Clients contacting local grassroots groups could then be seen and treated for free within these provider networks. In cases where needed care exceeded the cost of what volunteer providers could easily offer for free (e.g., expensive medicines, laboratory tests, x-rays, dental surgery, wheelchairs, or other medical aids), many grassroots groups collected or used donated funds to cover such individual expenses to the extent possible.

*“Usually, we just write down what we think is necessary and send the people to doctors willing to work with us without money. The money we have raised, we use for necessary treatments that are expensive, like x-ray or medication. That’s something we would pay. Sometimes it’s just a prescription and we send the patients to a nearby pharmacy and then just cover the bill.”* (local chapter of national network, active since 2015).

Other grassroots groups organized regular walk-in outpatient clinics staffed with volunteer physicians. Prior to 2015, these clinics mainly served undocumented migrants or citizens from Eastern and South-East Europe without health insurance coverage in Germany. Commonly these clinics offered follow-up care for chronic conditions, as well as psycho-social counselling to facilitate uninsured or undocumented individuals’ integration into the German health system.

*“We see a lot of people from Eastern Europe who can legally stay in Germany for six months. If they don’t find employment within six months, they don’t have access to welfare benefits provided by the German government. So, a lot of those people end up living in the streets, and they often are very sick.”* (local association, active before 2015).

#### Changes in operational roles in response to the 2015 refugee situation

From fall 2015 onwards, arriving refugees were officially assigned to reception camps and placed under the administration of municipalities. At the municipality level, different actors then coordinated the local emergency response. This way, existing – and later on newly forming – grassroots level relief organizations became involved in collaborating or supporting their municipalities in planning and implementing publicly coordinated efforts to organize and provide healthcare provision in these camps.

*“We assist in this humanitarian crisis by helping the health system. We are a part of this system at the moment. We are currently doing the work that should be done by others. We continue to do this work until the government starts to take up its responsibility to provide better healthcare for these people.”* (local chapter of national network, active since 2015).

Especially during this initial response, some municipalities fell behind in effectively providing shelter, social support, and healthcare to the often hundreds of refugees assigned to their reception camps. This frequently resulted in disruptions in consistently meeting refugees’ entitlements to essential healthcare. Interview respondents attributed such inefficiencies to the generally rather basic levels of crisis preparedness in most federal states and municipalities. In addition, the legal frameworks and bureaucratic hurdles defining the refugee healthcare processes often slowed down public actors’ ability to react in a timely manner. Many grassroots level groups therefore tried to identify alternative approaches less dependent on public organization and procurement.

*“We saw that setting up medical infrastructure was … difficult, let’s just put it that way. We weren’t very happy with the medical supplies that were dumped into the camp. This was done by another organization that did it for-profit. We had the feeling that the people living in the camp weren’t the focus. And these organizations paid for organizing the camp encountered the same problems over and over again. They knew what should be done differently, but they didn’t know how to provide this in a way that fit their allotted budget.”* (local chapter of national network, active before 2015).

In one narrative, one volunteer group that prior to 2015 had already been involved in organizing asylum seekers’ healthcare at their local level, directly sought a formal private-public collaboration with the local municipality in order to ensure effective medical care provision in a local camp. This group engaged in a contractual arrangement that allowed volunteer staff to be reimbursed for their time investment. In turn, the group guaranteed to consistently provide a level of medical care in the camp that meets humanitarian standards, but at much lower costs compared to other private actors.

*“This idea worked perfectly. We had all we needed and all they needed in this camp. And it was wonderfully organized. Just the necessary things – there was nothing extra, nothing fancy. We had many sponsors and companies that supported us, such as pharmacies. This money is not for us, but to lower the costs of refugee healthcare for the state. So, we are cheaper compared to a hired private for-profit partnership. This is a good combination.”* (local association, active before 2015).

### Role and contributions of non-governmental actors during the acute emergency response (fall 2015 to spring 2016)

Health needs of refugees changed somewhat over the course of the migration situation. So did the forms of support offered by non-governmental actors at the grassroots level. The period between fall 2015 and spring 2016 was primarily dominated by the continuing arrival of large numbers of migrants and their immediate health needs. With most refugees being allocated to reception camps across German towns and cities, various forms of interactions between local municipalities and grassroots level relief organizations emerged in planning and forming such a joint initial response.

*“The camp was officially run by the Red Cross. The camp clinic was run by a local primary care practice. They took this is a financial opportunity. You can make very, very good money with those camp clinics. The private providers operating clinics in those refugee camps, they got very, very well paid for this by the government*.” (local chapter of national network, active before 2015).

#### Healthcare provision in reception camps

Supporting healthcare provision inside camps, respondents frequently noticed discrepancies between the healthcare refugees were legally entitled to (i.e., health screenings, vaccinations, acute or emergency care, pregnancy-related care) and refugees’ existent health needs (i.e., non-acute traumatic injuries, post-traumatic mental disorders). To guarantee a service provision that stretches beyond these legally defined care entitlements, grassroots actors involved their volunteer provider networks as additional support structures for camp patients that requiring more than the legally entitled care.

*“Inside the camp, we were restricted by this law [i.e., Asylum Seekers Benefits Act]. But we said: let’s see that our 200 people here in the camp can get the necessary treatment for free, beyond of what is deemed necessary by law. As long as we could provide a service for free, we are no longer restricted in the care we provide. Only for very expensive care, we asked for an official treatment voucher. Otherwise, we just provided care for free. We encountered absolutely no problem. The municipality actually wanted us to provide good healthcare but was restricted to do so by law. We wanted the same thing. We just did it. And it worked.”* (local chapter of national network, active since 2015).

In some instances, camp organizers also actively approached volunteer provider networks to ensure their camp patients could access needed care by circumventing the bureaucratically more complex public health structures. While this usually produced more effective synergies between publicly organized camp clinics and grassroots level activist groups, few respondents perceived this as a negative consequence resulting from an overwhelmed public sector over-relying on the time and commitment of volunteers providing these free services, especially since municipalities often did not reimburse volunteer providers for their supportive non-governmental services.

*“We cannot just take up people we’re theoretically not responsible for. There have to be official structures in place to provide for this. We are simply a volunteer organization and don’t have funds to substitute healthcare provision at that scale. We cannot provide for something that has to be provided by the government.”* (local chapter of national network, active before 2015).

Interestingly, the experience of the one volunteer group that purposefully entered a contractual collaboration with its municipality in organizing camp clinics were particularly positive. Once refugee numbers increased, this arrangement allowed them to easily adjust their operational capacity by recruiting additional volunteer staff as their time commitments could be directly compensated. Further, this local collaboration gained additional media attention as being a more innovative approach to reception camp organization, which in turn resulted in publicity and a widening volunteer support for this joint project.

*“In 2016, we doubled our service hours. We just doubled our personnel. We now could see up to 120 patients a day. The increase in refugees was not really a problem, because we had this contract with the government which said we pay you by the hour. There was also a lot of public interest in our work. So, we presented our collaboration in a way to make clear that we want to help people professionally, but not for free. And so, we were able to recruit helpers, sponsors, and donations.”* (local association, active before 2015).

#### Healthcare provision outside reception camps

All respondents criticized the voucher system as a means to limit over-utilization of publicly financed healthcare for newly arriving refugees. As part of this system, German refugee law authorizes the state’s social services to issue vouchers for refugee patients to limit potential health costs during their initial 15 months in country. In the acute phase of the migration event, this voucher system and its overly bureaucratic processes produced otherwise avoidable delays in access to essential care.

*“The doctors are sometimes unsure what to do and then they become stricter than they could actually be. And for the refugees this results in a lot of running around. If they are sick, they first have to go to the social service to get a treatment voucher. They might wait there for two hours – if it’s a bad day – while being sick! Once the doctor decides more workup is needed but is unsure whether this is covered under the voucher, he has to write a treatment proposal, send it back to the social service or give it to the refugee to take it there for approval. Once the social service approves, the refugee can go to the doctor for this approved treatment. If a doctor treats without approval and the social service afterwards considers this treatment non-indicated, he doesn’t get reimbursed. Things like the costs of blood checks or x-rays. He’ll then end up paying for this out of own funds.”* (local chapter of national network, active since 2015).

Respondents observed how access to general practitioners, specialist doctors, or hospitals were complicated by the fact that many health system providers – at least during the initial crisis response – lacked sufficient technical and administrative familiarity with healthcare provision under this voucher system. This was especially the case with respect to compensation processes, as the voucher system will not reimburse physicians or hospitals for provided services not covered under immigration law. Thus, this system poses financial risks of refugee healthcare to any public provider treating a voucher patient. This potential financial risk produced some reluctance among providers to treat asylum seekers with some even refusing to see refugee patients at all.

*“A lot of doctors here in town didn’t know what they’re allowed to provide, what not, and whether they get paid. And sadly, we had this feeling that a lot of them simply preferred not to see people because they didn’t know whether they will get their money back and how the voucher system works. A lot of hospitals even kept the voucher forms, because they feared that otherwise they won’t get the money back from the municipality. As soon as the doctor or hospital kept the paper, the patients didn’t have anything in their hand to get additional medical support until the end of the quarter and had to collect a new voucher. Sometimes this voucher didn’t arrive in time and created unnecessary gaps in care provision.”* (local association, active before 2015).

A frequent observation was that many public providers did not receive adequate support or official guidance to better guarantee voucher patients received at least those essential services they were entitled to by law. This lack of adequate information flow between public fund holders (i.e., social offices as payers behind the vouchers) and public providers within the health system led to otherwise avoidable bottlenecks in refugee healthcare provision. Some grassroots groups therefore started advocacy and awareness activities to improve the flow of executing information towards those providers struggling with this form of service provision.

*“We would also go to doctors in the area close to the reception camps and ask them why they would not take on new refugee patients. We tried to inform these doctors about the voucher system, because most of the time these doctors did not know how to bill the city or state. So, most doctors did not even know about the whole financial logistics and processes related to the treatment voucher.”* (local chapter of national network, active before 2015).

### Role and contributions of non-governmental actors during the subacute crisis response (spring to fall 2016)

Following the closure of the Balkan Route, the influx of migrants decreased steadily. Many of the initially set up reception camps now started closing, as asylum seekers were relocated to smaller towns and communities outside bigger cities. At the same time, the vast majority of initially arrived refugees had now entered their asylum application process, during which they continued to be entitled to only limited publicly funded healthcare.

*“In March 2016, things changed as the Balkan route closed. And there were no longer any migrants arriving to Germany. So, we changed our focus towards supporting the accommodation centers by trying to integrate the asylum seekers into the national healthcare system.”* (local chapter of national network, active before 2015).

#### Barriers in accessing specialist healthcare

Interview respondents noticed an increase in the proportion of asylum applicants among their clientele. At the same time, asylum seekers’ overall healthcare needs shifted from more acute and camp-life related infectious conditions to include more specific chronic complaints often requiring specialized services, such as gynecological, dental, orthopedic, or psychological care. However, obtaining approval of such specialist healthcare under the voucher system was often highly bureaucratic and time-consuming.

*“I think there are quite a number of psychotherapists interested from what I’ve seen so far. A lot of them are quite motivated and would like to provide care. But it’s always a matter of what’s reimbursed and what not. There are some who say they do it on a voluntary basis. But of course, it would be nice if they would be paid for that. Sometimes a client can apply to be referred by the social welfare office.”* (local chapter of national network, active before 2015).

To offer such specialist care in a timelier manner, grassroots level actors often tried to expand their volunteer networks to include more specialists willing to offer their services for free. This, however, often proved to be quite challenging. For instance, some specialists in Germany are not widely available in every town or region (e.g., psycho-social care for patients suffering from post-traumatic stress disorders (PTSD)). Also, as specialist care is often more cost-intense, only few providers were willing to offer such services for free. In addition, as the refugee situation continued, healthcare providers became progressively less interested in actively supporting these grassroots level activities.

*“Our general practitioner comes every week. But what we actually need very much are more collaborating specialist doctors, like orthopedists or dentists. We would need them to say: Okay, twice a month, I am willing to treat somebody without being paid. But that’s very difficult, although it was very easy before the migrant crisis. Now they feel like they need a break.”* (local association, active before 2015).

With respect to psychosocial care needs, the legal restrictions posed a substantial access barrier to the emerging number of individuals suffering from posttraumatic mental disorders. While mental health disorders were often not detected or specifically addressed in the camps, they now became a dominant health need once asylum seekers were moved into accommodation centers located in smaller towns, as many PTSD-related disorders negatively affected individuals’ ability to successfully integrate.

*“Within these first 15 months, refugees usually do not get psychological support. Only in emergencies. I understand that resources are limited. But if someone has PTSD and has to wait, it’s quite difficult. Because the living conditions a lot of refugees are exposed to are quite stressful. They have already experienced quite some severe situations in their past, while fleeing, or because of war or conflicts. It’s difficult that they have to wait.”* (local chapter of national network, active before 2015).

#### Language as a healthcare barrier

While their unfamiliarity with the voucher system limited the accessibility to some public providers during the initial crisis response, providers now started to limit the number of refugees in their patient base for mere economic reasons. As public providers are largely compensated based on capitation and other bundled payments, many physician offices could not afford too many patients who likely required extra time investments, for instance due to language or communication barriers. Respondents observed that especially those physician offices near accommodation centers started capping the number of refugee patients these offices enrolled during a given quarter.

*“Doctors are afraid that too many asylum seekers are coming. If they say yes to one person per month, they’ll often end up with more. Then it becomes very hard for them to integrate these patients into their usual office structure, especially if too many people come who don’t speak the language. We have patients coming to our medical clinic that urgently need an ultrasound done or something, which we cannot provide here. It’s very hard to find a doctor who is willing to enroll them. We therefore need more specialists.”* (local association, active before 2015).

Interview respondents noticed that many municipalities lacked the infrastructural capacity and financial resources to offer qualified interpreter services that would have been needed for their administration as well as the local healthcare structures to improve refugee support mechanisms. Most grassroots level organizations therefore relied on their own established networks of volunteer interpreters to accompany refugees to physician or clinic visits. In some instances, however, these interpreter services were increasingly requested by local officials to accompany refugee clients also to local social or other administrative offices, limiting the capacity of interpreters available for healthcare visits.

*“One big challenge is interpreters. Now refugees are here for a while already, but of course they don’t speak the German perfectly. It’s absurd that they are expected to see a doctor on their own. A very high percentage of people have psychological problems. They all need interpreters to see a psychiatrist because it’s vital for consultation. And so far, our city has not really a solution how to deal with this.”* (local association, active before 2015).

Language and communication barriers were found to be extremely problematic with respect to psychosocial consultations, as these barriers limited the extent to which clients could be effectively assessed or treated. In this psychosocial healthcare context, translations were not only required to overcome the usual language and communication gaps, but to also to interpret any non-verbal communication or verbal hints relevant to a client’s mental health assessment. Arranging this level of qualified interpreter services was beyond the logistical and financial capacities of most grassroots organizations. Many respondents therefore had wished that, especially in view of the high need for psychotherapeutic care, capacities of a publicly funded interpreter infrastructure would have been put in place at an early stage.

*“The lack of qualified interpreters was a huge, huge challenge. In the beginning we used voluntary interpreters until we realized that we cannot work like this any longer. We needed qualified interpreters, especially qualified with respect to medical terms and especially in working with a psychiatrist or a psychotherapist. We looked for funds to pay qualified interpreters. That was one of the major challenges.”* (local chapter of national network, active before 2015).

#### Temporary coverage gap once transitioning out of the 15-month period

As already mentioned above, asylum seekers’ health costs are covered through public funds managed by the local social and welfare administrations during the initial 15 months in country. After this period – or once asylum had been granted – the administrative responsibility of financial health coverage is shifted to the local labor and employment administration (“job centers”). Respondents noticed that this shift in administrative responsibilities often caused temporary coverage gaps, as the bureaucratic processes involved were not sufficiently well timed to ensure a smooth transition from the voucher to the social health insurance system supported by the job centers. Individuals trapped in this bureaucratically produced coverage gap temporarily lost their financial health protection. For many then the only option to receive needed care had again to be met through the free clinics and provider networks offered by grassroots organizations outside the public systems.

*“They now get their electronic health card from the social service once they first arrive in Germany. Then after 15 months, they need to go to the job center to get a new health insurance card. Because it’s bureaucracy, it takes very long, it takes two months. So, during these two months, they have no coverage. And this happens all the time. Everybody is aware of this delay in bureaucracy, but nobody takes care of this. So, then these people come to us or to other volunteer organizations.”* (local association, active before 2015).

### Role and contributions of non-governmental actors during the early integration period (fall 2016 onwards)

The majority of asylum seekers now received their official decisions granting or denying their asylum. Health coverage for those whose asylum was granted transitioned into the social health insurance system. For rejected asylum seekers, health entitlements granted under immigration law so far, now ended.

*“When the first wave of rejection letters arrived, the mood in the accommodation centers changed. Particularly in those centers with a lot of asylum seekers from countries without a chance of getting asylum. These people had no perspective any longer, no right to work, not entitled to attend integration courses. Only because they came from a safe country-of-origin.”* (local chapter of national network, active before 2015).

#### Rejected asylum and mental health

Individuals who received a negative asylum decision were now required to self-pay their care. Those suffering from medical or mental health problems that made it impossible to return to their countries of origin were now required to obtain an “unfit-for-travel” certificate to delay or remove their obligation to leave Germany due to health reasons. Given the recent amendments to refugee law, these certificates could now only be issued by a medically trained provider. As many rejected individuals had no longer free access to public providers, grassroots organizations played again a central role in supporting this process by identifying medical providers willing to issue such certificates.

*“What we did is to setup a system of cooperation with psychologists, psychiatric doctors, to issue certificates for clients about their psychological health evaluation. Especially for people threatened by deportation, because deportation is one of the main factors worsening health after psychological trauma. The rules and laws now get increasingly stricter, stating that somebody suffering from PTSD is still fit for deportation. So, now our whole system is basically obsolete.”* (local chapter of nation-wide organization, active before 2015).

The amended law no longer allowed psychologists (and other non-medical healthcare providers) to issue “unfit-for-travel” certificates. Especially for individuals suffering from mental health disorders, the added legal restriction made it impossible to obtain a certificate from their treating psychologists. This drastically increased the need for medically trained mental health experts, such as psychiatrists or medically trained consultants. One grassroots group therefore set up a local system where they enabled direct collaborations between treating psychologists and volunteer medical providers to be able to issue “unfit-for-travel” certificates more easily based on the indication of the treating psychologist.

*“We now see more people in need of an unfit-for-travel certificate. We started a cooperation with the Psychosoziales Behandlungszentrum [i.e., psychosocial treatment center] because according to the “Asylpaket 2” since July 2016 [*i.e.*, revised regulatory framework containing the Act for the Introduction of Expedited Asylum Procedures], psychologist can no longer certify a traumatized refugee’s unfitness to travel as a means to prevent deportation. Their assessments are no longer accepted by law, as they are not medical doctors by training, they are “only” psychologist. That’s why we are now going to start this cooperation to find some suitable solution: we have the medical doctors; they have the psychosocial therapists.”* (local chapter of national network, active since 2015).

#### Vulnerable groups

Life in an accommodation center only allows limited privacy and often exposes individuals to social conflicts of other residents. Especially for women and children, as well as unaccompanied minors placed under the authority of the state, this posed additional risks to their mental and social health.

*“Our focus has changed. We’ve stopped the pediatric consultations. There was just no need anymore, as the integration of kids into the national healthcare system was a lot easier compared to adults. So, right now we focus mostly on accommodation centers housing a lot of young boys aged between 18 and 30 with no prospects of legally staying in Germany. They all received their negative asylum decision. Their asylum applications have been rejected.”* (local chapter of national network, active before 2015).

Refugees with recognized asylum now had official entitlement to the full range of benefits and public social support offered to German citizens. Non-governmental actors therefore shifted their focus on the remaining, most vulnerable refugee populations still prone to greater health risks, such as unaccompanied minors, women, and undocumented migrants.

*“The worse the mood, the more people start pondering, just sit around and have negative thoughts. Old trauma can come up. We now see lots of psychological cases and these cases will increase further. We do a lot of awareness work on how living conditions in those centers actually make the psychological conditions of asylum seekers worse. They have 80 people sleep in one huge old office room. It’s always loud. It’s not safe for women. Poor living conditions. We try to get more media attention specifically on the situation of women who are here without their husbands, single women, single mothers with their kids.”* (local chapter of national network, active before 2015).

### Lessons learned

Respondents were asked to reflect on their experiences to identify any lessons learned that could be relevant for the German state or any public or private stakeholders when planning or preparing their response to a future migration event. In the following, we present these propositions within five wider themes.

#### Communication of healthcare information

Respondents perceived the public efforts in disseminating health information to asylum seekers as quite limited, especially with respect to essential content and to the range of translated languages. Key health messaging often lacked the needed detail on how to guide a foreign person in effectively accessing and navigating the healthcare system. This was especially the case with respect to the voucher process, where even health providers themselves were frequently not able to provide this guidance to their patients.

*“We offered information sessions where we prepared a PowerPoint presentation about how things work. And we explained the difference between the voucher and the health card. But this was directed to people who already lived here for some time. The people who newly arrive here, I don’t know how information works for them. I hope they will get the information from the social workers in the camp where they stay. It should be like this. I mean, this is the official channel.”* (local association, active before 2015).

Further, available information was frequently only offered in few languages, with the set of language translations not sufficiently targeting the geographical or cultural backgrounds of local refugee populations. Thus, public messaging efforts remained often relatively limited in ensuring essential information could be easily accessed and used.

*“The German health system is difficult to understand. How is the system structured? When does one call a general practitioner? You have to go there and be on time. And it’s normal having to wait for an appointment with an eye doctor. We do this education now in different languages. In Farsi, Arabic, and Russian. We worked on this information campaign together with the city’s health authorities. They are very, very grateful to us because we do a lot of these things that they should do but aren’t doing.”* (local association, active before 2015).

#### Interpretation of refugee law

An observation shared by most interview respondents was that both public administrators and healthcare providers were little prepared to fully guarantee that every refugee could access and receive the minimum care they were legally entitled to. Existing legal and bureaucratic frameworks were often not sufficiently clear in assigning administrative responsibilities between public agencies. Respondents therefore proposed to consider alternative structures to effectively improve the organization and collaboration between the various actors involved in refugee healthcare.

*“We are very much in favor of having so called “Medi-Points” assigned to refugee camps. To have an integrated Medi-Point that’s financed by the state or city where asylum seekers can go and get all the help they need. And where interpreters are provided. This has been done in other federal states, but not here. Apparently, there’s no money for that. The state is unwilling to pay for such services, but instead asking for volunteer doctors to do provide this. This is not sustainable and it’s not how it should be, because we should not supplement the state.”* (local chapter of national network, active before 2015).

Especially grassroots activists organized as local chapters of wider network organizations gained wider insights in how local response efforts differed across jurisdictions or regions. This allowed them to more directly compare how similar bureaucratic hurdles had been dealt with across jurisdictions. Such differences in approaches often related to the often variable legal interpretation and local implementation of refugee healthcare regulations across regions and municipalities. Respondents also pointed out that those states or municipalities that pursued a more refugee-focused interpretation usually more easily identified more integrative approaches to responding to locally encountered problems.

*“There is a great example in Frankfurt. Frankfurt has no social offices anymore, but instead a health office where everybody can come and ask for medical assistance. They also focused on incorporating different immigration regulations more comprehensively by offering a so-called “humanitarian consultation hour”. And they don’t check one’s identity, they don’t check one’s nationality, and they don’t check one’s name.”* (local chapter of nation-wide organization, active since 2015).

#### Replacing the voucher system

The voucher system and its operationalization were criticized in each of the recorded interviews. Especially the administrative approach in using social service offices as key intermediaries between patients and providers was seen as a questionable extra layer of complexity and bureaucracy. Respondents experienced the voucher system as failing its intended purpose as a financial control mechanism.

*“In my opinion one improvement is that in some parts of Germany they introduced now the electronical health card [instead of the treatment vouchers]. I think this is in response to the increasing numbers of refugees and their need in seeing a doctor. The whole voucher bureaucracy overwhelmed many people. So, this situation might have triggered policy makers to allow these electronical cards which make it easier for people.” (local chapter of national network, active before 2015).*

At the time of our interviews, some federal states and municipalities already started replacing the voucher system with electronic health cards. Similar to the electronic insurance card used by the social health insurance system, physician offices were now given a stronger role as initial contact point for asylum seekers and as their gatekeeper to the health system. By having the processes in refugee healthcare provision more closely aligned with those common to the social healthcare system better facilitated the clinical and financial management of asylum seeker patients during the 15-month period.

*“I still think the best solution is the electronic healthcare card for refugees. Right at the start of the asylum application. This is the only way to create unbureaucratic, cheap access to healthcare. And we know it’s cheaper! It’s this political argument that says: We don’t want this, because we’re afraid that this might be a pull factor. But we know that this card offers the best access to healthcare. It would cover psychological health, physical health, and to some extent even social health. But it’s just not done everywhere. It’s not done because it’s very unattractive within conservative political circles.”* (local chapter of nation-wide organization, active before 2015).

#### Mental healthcare

Many respondents gained the impression that the publicly organized health system was little preprepared in effectively handling the high prevalence of mental health problems emerging among refugees from conflict zones. The limited capacity to offer better specialized posttraumatic care was largely also a result of the overall limited availability of such specialized mental care providers within the German health system.

*“We have big, big problems when it comes to providing psychiatric therapy to refugees. And this actually shows how difficult the language barrier is. They have almost no access to psychiatric healthcare because of the language barrier. We still have almost no options to provide mother-tongue therapy for refugees. This is an indicator of how bad this interpreter system is developed, it’s still underdeveloped. Completely underdeveloped, in almost every aspect.”* (local chapter of nation-wide organization, active before 2015).

In the near future, violent conflicts like the Syrian civil war are likely represent a main cause of mass displacements similar to the events in 2015. Psychological traumatization therefore will remain a major disease burden among future refugee populations. Many respondents therefore consider the improvement of mental healthcare capacities, especially with respect to PTSD as a key element in future crisis preparedness.

*“A lot of people have PTSD or other psychological disorders or mental problems which simply were not recognized at first. Of course, every refugee undergoes a health assessment after they arrive here. But they only check whether a person has tuberculosis or is fully vaccinated. There’s no real screening for psychological problems. There should be a screening tool like a questionnaire that could be used for refugees once they apply for asylum. Just somebody who asks them if they sleep well, if they eat well, and all these questions. But that would require extra people to do the job and a lot of time and would be difficult to finance*.” (local chapter of national network, active before 2015).

#### Undocumented migrants

Given the increasing legal restrictions on political asylum, many interview respondents expect the numbers of undocumented migrants living in Germany to further grow. Although the peak of migration has flattened, the number of undocumented migrants still living in Germany probably continues to raise as rejected asylum seekers might decide to stay illegally. Unfortunately, current legislation makes it nearly impossible for these undocumented migrants to safely access healthcare without risking legal repercussions or deportation.

*“We should implement a system in which a lawyer and/or social worker sits in an office and an undocumented person can approach without fear of repression. And together they will figure out options to access healthcare. They might take legal actions against an insurance company. They might directly communicate with hospitals or with the city council, or so. This is the first step to provide healthcare for everybody by default.”* (local chapter of nation-wide organization, active before 2015).

Public officials in Germany are legally required to disclose information about undocumented migrants to the immigration office. Undocumented migrants therefore often avoid seeking care from health providers within the public system. For them, healthcare provided through grassroots and other humanitarian organizations is often the only safe option. Interview respondents thus proposed the introduction of alternative approaches to offer care to this patient group to not only ensure financial access to essential services, but also a safe and protective environment.

*“I think there should be access to the health services for everybody, including people without papers. In Thuringia, for example, they introduced the “Anonymer Krankenschein” [*i.e.*, anonymous health voucher]. They can obtain this voucher that grants access to health services without disclosing their personal data. They can get it from the social office. It’s a form that entitles to treatment from a doctor without telling your name. It’s anonymous. In case you don’t want to disclose your name because you fear deportation. That’s a first step.” local chapter of national network, active since 2015).*

## Discussion

Humanitarian relief organizations are key actors in defining and organizing the emergency response to humanitarian crises. Their work and contributions, however, are often more noticeable in situations of weak governance and inadequate public infrastructure [2, 18]. Little research exists on the contributions of humanitarian actors in the context of politically stable nations facing a domestic humanitarian emergency. In examining the 2015 refugee situation with respect to Germany’s humanitarian response, this study explored non-governmental humanitarian actors at the grassroots level to explore their role and contributions to the country’s efforts in providing refugee healthcare. Being the first study with an explicit focus on grassroots level activists, this study offers additional perspectives on Germany’s crisis response.

### Immediate emergency response

Based on our findings, the initial role of interviewed grassroots actors was that of first responders within the context of publicly organized refugee camps. This included, for instance, the organization of medical care provision in camps within an ad-hoc private-public partnership between a locally active grassroots organization and its municipality. Frequently, grassroots groups also complemented publicly organized response strategies to guarantee that basic health entitlements of refugees could be met. As non-governmental actors, many grassroots activists further ensured that essential care could be provided beyond legally imposed restrictions and bureaucratic regulations.

Some of our findings largely align with existing evidence on bottlenecks in the provision of refugee healthcare, such as the legally imposed restrictions to essential healthcare or the limited accessibility of information on navigating the German health system prior to 2015 [25]. To this extent, our findings echo how these political restrictions to universal healthcare for migrants further complicated the public system’s ability to develop a more streamlined provision of refugee healthcare in 2015. Furthermore, not only the organization but also the quality of healthcare provided to refugee populations varied largely depending on how a municipality decided to interpret or apply the underlying legal script [9]. A result of the vague language used in refugee law was is found in the use of variable definitions for “non-acute” conditions or in the implementation of different clinical protocols with respect to infectious disease screenings or vaccinations [11, 26].

In the absence of a unified response plan, healthcare provision inside camps across Germany followed different organizational and provider models [27]. Based on our findings, the organization of camp clinics around available resources to meet required outputs depended largely on municipalities’ ability to collaborate with local for-profit and not-for-profit actors. Our findings suggests that without direct or indirect involvement of non-governmental actors and reliance on their grassroots level networks to offer free care, publicly organized healthcare provision would often not have been adequate in guaranteeing camp residents’ access to entitled essential care, especially during the immediate response phase.

### Access to publicly funded healthcare

Roles and contributions of grassroots organizations changed once refugee healthcare could be more comprehensively provided by the publicly funded health system. At that point, activists’ roles transitioned from first responders towards refugee advocates supporting patients in having their needs met and their rights respected within the context of a voucher system that not only complicated access to care, but also limited public providers engagement in refugee healthcare provision. Similar to our findings, earlier studies already described the German voucher system as highly bureaucratic with the potential to deny essential healthcare in situations where basic medical care could otherwise have been easily provided [9, 15, 16, 28]. Further, earlier studies also reported on instances of delayed or denied access to entitled care given the limitations providers faced in instances where necessary medical care had been beyond what the law considered as indicated. Our findings further suggest that not only the implementation but also the transition out of the temporary voucher system resulted in situations of limited access or healthcare coverage.

One important finding of our study is the relative unfamiliarity of public healthcare providers, both physician offices and hospitals, with the financial mechanism related to patient care under the voucher system. We identified only one other study, which assessed medical care providers’ familiarity with the specifics regulating the compensation of healthcare provision to refugees in Germany. Based on this study, only 68% of doctor offices and 36% of hospital-based physicians felt sufficiently aware of the reimbursement modalities for medical care provided to refugees in early 2016 [29]. As illustrated in some interview narratives, unfamiliarity with regulatory processes and the potential loss of revenue led to instances where voucher patients were not seen by public providers and instead referred to free clinics operated by grassroots volunteers outside the public system.

Besides advocacy during the subacute crisis response, many grassroots organizations continued to complement or back public healthcare provision, for instance, by providing free interpreter support to patients, to providers, as well as to public administrators. While cultural and language barriers are known challenges to how healthcare can be provided and utilized by a certain population group, our findings suggest that language barriers were most challenging with respect to the provision of mental health and PTSD care. Based on recent data, the actual prevalence of PTSD among refugees in Germany might have been as high as 80% depending on a refugee’s home country and flight context [30, 31]. In the 2015 events, this challenge was further aggravated by the unexpected or underestimated PTSD burden among refugees, especially among those with Syrian origin [32].

### Financial health protection for refugees

Gaps in the response coordination, language barriers, or the underestimation of specific disease burdens all contributed to deficits in the provision of refugee healthcare. However, the underlying systematic problem the public system faced in adequately responding to these health needs largely stemmed from the imposed restrictions in refugees’ financial health protection as defined in the Asylum Seekers Benefits Act (i.e., limited financial healthcare coverage for the initial 15 months in country) and the Act’s operationalization in form of the voucher system. Our findings suggest that grassroots organizations replaced to a large extent those elements of financial protection the Benefits Act withdrew by offering free care for health services not considered essential by law.

Based on the interview narratives, it appears that in most instances the provision of free medical services was sufficient to enable refugee patients to receive timely and unbureaucratic basic care. Existing evidence even suggests that in jurisdictions where the voucher system was replaced, primary healthcare utilization improved, the use of more costly emergency care reduced, and healthcare costs incurred during the 15-month period remained overall lower [33, 34]. As of our knowledge, no data exists on the total public costs saved (either by the indirect shift in healthcare provision to volunteer providers, or with respect to negative health outcomes prevented by these support services) attributable to the direct or indirect involvement of non-governmental actors in the provision of refugee healthcare in 2015, especially considering the financial and non-financial contributions of volunteers at the grassroots.

### Future research needs

This study also identified additional aspects related to refugee healthcare provision that we were not able to explore further and therefore might benefit from additional research in the future. For instance, current preparedness and communication strategies fail to adequately address how healthcare restrictions should be implemented or operationalized to ensure both legal and ethical expectations are consistently met. Further research to also understand experiences and decisions of local administrators and healthcare workers in a context where health entitlements are restricted not so much because of resource limitations, but largely due to a politically determined exclusion of a non-represented population group, might therefore yield useful insight to a wider discussion on refugee law implementation in Germany.

In addition, more research will be needed to improve the predictability of migration events reaching Europe and the expected healthcare needs of defined migrant or refugee populations during the first months or year of their stay. Applying and incorporating such forecasts into state and local preparedness plans might not only allow better predictability of the expected disease burden and a needs-focused preparation or activation of those sectors in the health system best suited to address this burden (including the provision and deployment of necessary care funds), but also an “event-specific” legal definition of what basic care needs should entail.

Further, our study revealed that there might be quite a few municipalities that developed and implemented local or regional care models for refugees, such as concepts of integrated care, with the potential to streamline and improve the provision of healthcare and social services to refugee populations. Exploring and studying these different approaches to refugee law implementation and its operationalization might yield valuable insights in how refugee healthcare could become more efficient, less costly, and more effective. Identifying and exemplifying such success models could help other communities to better prepare their health and administrative infrastructure for future migration events like those experienced in 2015.

Lastly, our study was unable to sufficiently represent experiences and insights from organizations operating in the new German states. To better understand whether the circumstances and challenges identified in our study can be similarly applied to other parts of Germany, future studies should try to specifically recruit and include informants active in the new states.

### Limitations of this study

Our study has some limitations. First, our study only includes non-governmental organizations that had a visible online presence at the time of sampling, and we might have excluded eligible study participants without the means or know-how to invest in such an online presence. This consequently might have resulted in the omission of additional potentially valuable information such participants might have contributed. To control this bias, we therefore added a snow-ball component to our recruitment strategy which involved that ever person contacted and interviewed was further asked about additional actors active in their geographical or care-specific area, we tried to control this bias to the extent possible. Without an official roster of active organizations available to us, our initial online search identified 77 entities covering a wide range of different organizational types (i.e., associations, groups, or individuals), sizes, and locations. Thus, we felt sufficiently confident that this potential selection bias was minimal.

Second, the response rate after initial contact was relatively low, which might have biased our findings as experiences from non-governmental actors unwilling or unable to participate in our study could have differed substantially. Still, we consider our final sample sufficiently reflective of our selection criteria as we found the information content captured by our interviews to have reached saturation. As noted in the Methods, about half of identified organizations did not respond after two contact attempts by email. In reviewing key characteristics available to us based on our online research, we could not identify a clear difference in patterns between contacted non-responders and those respondents declining participation. We therefore assume that other characteristics unknown to us, such as an organization’s communication capacity or by-laws regarding organizational approval of research participation might have affected this outcome. Hence, we face difficulties to clearly define the extent of self-selection bias reflected in our final sample. For this reason, we consider the generalizability of our finding limited beyond the described group of respondents interviewed. For future studies using a similar recruitment approach, we therefore suggest to also include other means of contacting, such as phone calls and written postal mail, as this might not only reduce the proportion of non-responders but might also decrease the proportion of refusals.

Third, while not intended, we were unable to include grassroots organizations operating in the new German states. While the media frequently reports on less favorable attitudes towards foreigners or refugees in the East, research demonstrates that the general support for refugee integration in Germany does not differ geographically [35]. Further, the political and legal frameworks and their implications on refugee healthcare in 2015 did not significantly differ between new and old German states [36]. We therefore do not assume that the absence of respondents from the new German states might have considerably biased our overall findings. Lastly, interviews were conducted in English, not in respondents’ native German language. Limited language fluency might have restricted the ability of some respondents to express their perceptions or experiences to the extent they otherwise would have.

## Conclusions

The 2015 mass displacement required German states and municipalities to develop a humanitarian emergency response that meets the healthcare needs of refugees assigned to their jurisdictions. Non-governmental humanitarian organizations at the grassroots level played a key role in contributing to this locally organized response. With respect to refugee healthcare provision, we identified several contributions by these non-governmental actors that supported or complemented public efforts. In offering free medical care, grassroots level volunteers were able to ensure essential healthcare to refugees beyond their legal healthcare entitlements. Through advocacy and awareness activities, grassroots activists ensured that refugees could be guaranteed access to entitled healthcare provided within the public health system. Grassroots organizations further provided interpreter support essential for the direct interaction between patients, providers, and public administrators. Lastly, the direct and indirect involvement of grassroots organizations not only ensured access to care, but also provided a substantial contribution to refugees’ financial health protection in instances where public regulations or bureaucratic practices failed to do so.

## Supplementary Information

**Additional file 1.**

## Data Availability

The datasets used and/or analyzed during the current study are available from the corresponding author on reasonable request.

## Abbreviation

PTSD: Posttraumatic stress disorder
