# Supplementary material for: “We assist the health system doing the work that should be done by others” – a qualitative study on experiences of grassroots level organizations providing refugee health care during the 2015 migration event in Germany
Source: BMC Health Serv Res. 2022 Mar 7;22:309. doi: 10.1186/s12913-022-07683-2 (PMC8900432; doi:10.1186/s12913-022-07683-2)
Supplement: Supplementary file 1 — Additional file 1. [file 12913_2022_7683_MOESM1_ESM.pdf]

UniversitätsKlinikum Heidelberg

INTERVIEW GUIDE

Title of Study: Health Coverage for Refugees in Germany - A Qualitative Study of the Role and Challenges of Non-Government Organisations during the “Refugee Crisis”

Preamble:
The aim of this study is to better understand how NGOs in Germany have responded in the provision of refugee health services during the recent increase in migration.

You have been invited to participate in this study because of your organisation’s active role in providing health-related goods and/or services to asylum seekers and/or refugees. I encourage you to share your experiences and thoughts about any recent changes in responding to health needs for this group. In addition, I am also interested in your opinion on developments within the German health sector.

I have received a written consent from you, which outlined the procedures and details of this interview. As a kind reminder, I will be audio recording our discussion, though you may request at any point to stop the recording. Lastly, the interview should not take more than 60 minutes of your time. If there are any further questions, please share them with me now before we begin.

Questions:

<u>Background Information</u>

1. Can you please state your role and number of years you’ve been at this organisation?

2. And to get more background information about your organisation, can you tell me the staff size and number of years your organisation has been in operation?

<u>Organisational Operations</u>

3. Can you please describe your current role in providing health-related services to asylum seekers and refugees?
   Probe: *Describe the good or service? What is your coverage area? How do clients learn about you? Who in particular is the target demographic? How many clients?*

4. And has your role needed to change because of the recent increase in migration to Germany (or even leading up to it)?
   4.1. If yes, in what ways?
   Probe: *Interest in utilisation of services? Financial and/or resource support? Interest in involvement and/or employment?*
   4.2. How are/were you able (or not able) to make these changes?
   Probe: *Describe experience - support or challenges?*

5. Can you describe how you were able to understand the gaps and health needs during this time (in order to respond)?
   Probe: *Data/statistics internally and/or externally monitored? Sources for updates in news & policies? Sources for paying attention to other efforts within the health sector? Perceived impact and/or benefits?*
   5.1. And for ongoing understanding of the gaps and changing health needs, can you comment on what would be an ideal process (or improvements)?
   Probe: *And how would this information or process be helpful to you?*

<u>Health Sector Developments</u>

6. If we now look at the German health sector, what did you feel were strengths or successes in the response to providing healthcare services?
   Probe: *Describe the process and evolution.*

7. And any ignored areas or remaining gap(s) in need of addressing?
   7.1. How might we solve this?
   Probe: *Solutions? Ideas? Thoughts? Partnerships/collaborations? And why would it work?*

8. Last question — Would it be advantageous if NGOs had an even larger role in providing refugee health services?
   Probe: *If yes, why? How to make possible?*

Closing Remarks:
Thank you once again for participating in this study. Your responses have been very informative and will help contribute to understanding better the healthcare services available to asylum seekers and refugees in Germany.

I take the time once again to remind you that everything you have shared with me, including your participation in this study, will remain confidential. Should you have any reservations about continued participation at the conclusion of the interview or thereafter, please do no hesitate to reach out and express your concerns; immediate and appropriate action will be taken to respect your wishes and privacy. Alternatively, if you would like to follow-up with more commentary, or, have further questions, please also get in touch with me. Thank you!

[Off-Tape - Snowball]:
Are there any other individuals or organisations in this field that you think I should consider inviting to the study?
